# In vitro investigation of cytotoxic and antioxidative activities of *Ardisia crispa* against breast cancer cell lines, MCF-7 and MDA-MB-231

**DOI:** 10.1186/s12906-018-2153-5

**Published:** 2018-03-12

**Authors:** Muhammad Luqman Nordin, Arifah Abdul Kadir, Zainul Amiruddin Zakaria, Rasedee Abdullah, Muhammad Nazrul Hakim Abdullah

**Affiliations:** 1 Department of Clinical, Faculty of Veterinary Medicine, Universiti Malaysia Kelantan (UMK), Pengkalan Chepa, 16100 Kota Bharu, Kelantan Malaysia; 2 Department of Veterinary Preclinical Sciences, Faculty of Veterinary Medicine, Universiti Putra Malaysia (UPM), 43400 Serdang, Selangor Malaysia; 3 Department of Biomedical Science, Faculty of Medicine and Health Sciences, Universiti Putra Malaysia (UPM), 43400 Serdang, Selangor Malaysia; 4 Department of Veterinary Laboratory Diagnostics, Faculty of Veterinary Medicine, Universiti Putra Malaysia (UPM), 43400 Serdang, Selangor Malaysia

**Keywords:** Cytotoxic, *Ardisia crispa*, Hydromethanolic, Breast cancer, Antioxidant

## Abstract

**Background:**

*Ardisia crispa Thunb*. D.C is used mostly in some parts of the Asian region by traditional practitioners to treat certain diseases associated with oxidative stress and inflammation including cancer and rheumatism. In Malaysia, it is popularly known as ‘Mata Ayam’ and local traditional practitioners believed that the root of the plant is therapeutically beneficial.

**Methods:**

The cytotoxic effect of hydromethanolic extract of *A. crispa* and its solvents partitions (ethyl acetate and aqueous extracts) against breast cancer cells were evaluated by using MTT assay. The cells were treated with concentration of extracts ranging from 15.63 μg/mL- 1000 μg/mL for 72 h. The quantification of phenolic and flavonoid contents of the extracts were carried out to determine the relationship between of phytochemical compounds responsible for cytotoxic and antioxidative activities. The antioxidant capacity was measured by DPPH and ABTS free radical scavenging assay and expressed as milligram (mg) Trolox equivalent antioxidant capacity per 1 g (g) of tested extract.

**Results:**

The hydromethanolic and ethyl acetate extracts showed moderate cytotoxic effect against MCF-7 with IC_50_ values of 57.35 ± 19.33 μg/mL, and 54.98 ± 14.10 μg/mL, respectively but aqueous extract was inactive against MCF-7. For MDA-MB-231, hydromethanolic, ethyl acetate and aqueous extracts exhibited weak cytotoxic effects against MDA-MB-231 with IC_50_ values more than 100 μg/mL. The plant revealed high total phenolic content, total flavonoid and antioxidant capacity.

**Conclusion:**

The response of different type of breast cancer cell lines towards *A. crispa* extract and its partitions varied. Accordingly, hydromethanolic and ethyl acetate extracts appear to be more cytotoxic to oestrogen receptor (ER) positive breast cancer than oestrogen receptor (ER) negative breast cancer. However, aqueous extract appears to have poor activity to both types of breast cancer. Besides that, hydromethanolic and ethyl acetate extracts exhibit higher TPC, TFC and antioxidant capacity compared to aqueous extract. Synergistic effect of anticancer and antioxidant bioactives compounds of *A. crispa* plausibly contributed to the cytotoxic effects of the extract.

## Background

Mammary cancer is a type cancer that arises from the mammary glands. In humans, it is commonly known as breast cancer due to the anatomical location at the breast. In animals, it is still known as mammary cancer. Globally, breast cancer is the most frequent cancer amongst women as it accounts for high mortality rate especially in non-developing countries due to late diagnosis and population increase. Interestingly, breast cancer also can develop in males, though relatively very rare (less than 1%) [1] and the pathophysiology remains uncertain [2, 3].

Since 2003, there have been several new updates of breast cancer classifications. However, until now, the fundamentals of breast cancer classification are still based on pathology and molecular biology [4, 5]. Evidently, pathological classification is based on characteristics seen under light microscopy of biopsy specimens. In 2003, the report from World Health Organization (WHO) stated that there are 20 major tumour types [6]. The debate on the classes of mammary cancers is still on [6]. However, a majority of the accepted positions are that most mammary cancers are derived from epithelium lining ducts and lobules [5]. Thus, these mammary cancers are pathological classified as ductal or lobular carcinoma [6]. Besides, as part of pathological classification, the presence of pathological grades such as the presence of acinar, glandular and pleomorphic in mammary cancers morphology also would be able to determine the prognosis of the patient [7, 8]. Conversely, the biological classification is mostly based on endocrinology gene expression, which are oestrogen receptor (ER) positive/negative, progesterone receptor (PR)-positive/negative, human epidermal growth factor (HER2)-positive or HER2-negative type of mammary cancer [5, 6]. However, there is still space to study the molecular biology classification of mammary cancer due to some overlapping amongst immunohistochemistry surrogate and many molecular classes and subtypes [4]. However, there are two types of breast cancer cells that have gained interest amongst researchers. They are the oestrogen receptor (ER) positive breast cancer, MCF-7 [9] and oestrogen receptor (ER) negative breast cancer, MDA-MB-231 [10].

Since ancient immemorial times, medicines from herbal and natural products were widely used in every culture throughout the world. Medicinal plants generally known as herbs played significant roles in the development of drugs and the outcomes have been promising. Therefore, in the view of exploration for an alternative medicine, particularly breast cancer studies, local Asian plant named, *Ardisia crispa* (Thunb.) A. DC plant was selected due to evidence that the plant exhibits anti-inflammatory activity that can be relevant to anti-breast cancer. More so, anti-inflammation is often associated with inhibition of angiogenesis [11], which co-jointly regulate the activation of cell chemotaxis, migration, and proliferation, and thus has the potential of suppressing tumour growth and metastases. Hence, the inhibition of angiogenesis is one of the most promising strategies in the development of novel anti-cancer therapies, and in the treatment of other human diseases associated with angiogenesis.

Phytochemical analysis from the leaves extract of *A. crispa* showed the presence of many phytochemicals compound such as flavonoids, phenolics, saponins, tannins, terpenoids, and steroids [12]. In a previous study by [11], it was revealed that the root of *A. crispa* contains various phytochemical compound such as phenolic, flavonoid and saponin when hydroethanolic is used as a solvent system. This plant extract also showed several biological activities such as anti-inflammatory and anti-hyperalgesic [11, 13], antipyrexic [14] and antiulcer [15]. Besides that, *A. crispa* have been reported to possess cytotoxic effect against human liver cancer (HepG2), skin cancer cells [16, 17] and mouse mammary cancer (4 T1) [12]. It is believed that the plant has anti-inflammatory properties by inhibiting angiogenesis process. It is also proposed that anti-inflammatory mechanism could partly involve in the anticancer activity. Therefore, plants which exert anti-inflammatory activity will usually exert anticancer activity.

Oxidative stress is one of the pathways of carcinogenesis and the phenomenon is often associated with inflammatory cells. The connection between antioxidative and anticancer activities has been widely subjected to extant empirical endeavours. *Ardisia crispa* is an evergreen flowering plant that belongs to the family *Myrsinaceae*. It is widely distributed over Asian regions including Malaysia. Local Malaysian people know this plant as ‘pokok mata ayam’ or ‘pokok mata pelanduk’. Traditionally, the root extract of *A. crispa* is believed to be useful in the treatment of several human ailments such as liver cancer, swelling, rheumatism, cough, fever, diarrhoea, broken bones, women dysmenorrhoeal, respiratory tract infections, and traumatic injuries.

Thus far, no empirical submission has been reported on the cytotoxic and antioxidative properties of any *Ardisia* species against breast cancer cell including *Ardisia crispa.* This study was conducted with the intention of discovering the true potential of the local herbs for anti-breast cancer activity which could perhaps reduce the side effects of current treatment. It could also be used synergistically with the available one to subsequently improve their pharmacological and toxicological effect and prognosis of the treatment. Furthermore, complementary methods such as using herbs or vitamins to treat cancer or relieve side effects of cancer is not something new.

## Methods

### Collection and identification

**Fig. 1:**
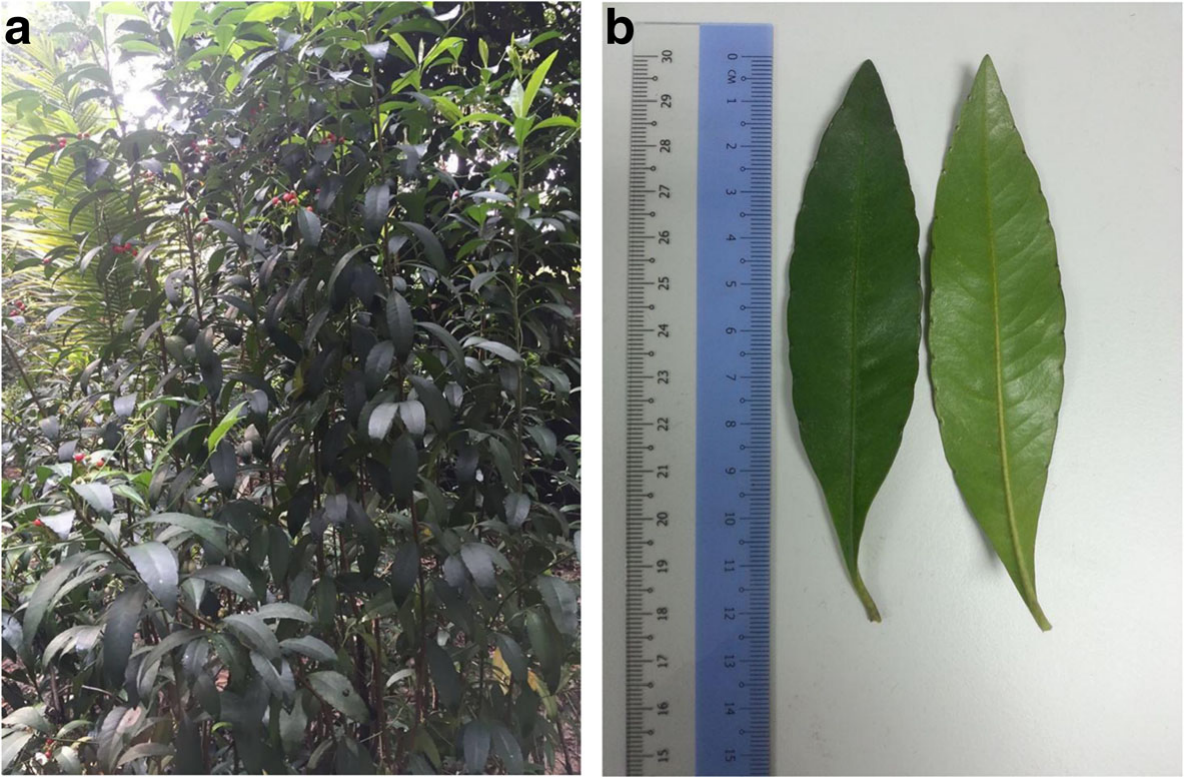
*Ardisia crispa* Thunb. D.C. Photo was captured at the Agricultural Conservatory Park, Institute of Bioscience, Universiti Putra Malaysia. **a** plant **b** leaves

*A. crispa* fresh leaves were collected from Biodiversity Unit, Universiti Putra Malaysia (UPM). The plant samples were certified by botanist of the Institute Bioscience (IBS), UPM, Serdang, Selangor, Malaysia by comparing with a deposited voucher specimen (SK 2834/15) from Herbarium of Natural Products, IBS, UPM. The appearance of *A. crispa* is shown in Fig. 1. The leaves were cleaned and then dried in oven at 37 °C for a week.

### Extraction of plant leaves

The dried leaves of *A. crispa* were pulverized to become tiny particles by WARING commercial blender model HGB2WTS3. Approximately 400 g tiny particles of plant leaves were soaked in methanol: distilled water (80:20, *v*/v) at room temperature for 72 h in a 4000 mL glass conical flask. The ratio between sample and solvent was 1:20 (*w*/*v*). The mouth of the flasks was wrapped with aluminium foil to prevent evaporation of the solvent which was under continues daily shaking for three consecutive days. The mixtures were filtrated through cloth filter and cotton wool to separate solvent-containing extract with the pulverized leaves and continued filtered with Whatman No. 42 filter paper to glean cleaned solvent-containing extract. Then, the pulverized leaves were collected and subjected to similar extraction process for another two times. The solvent-containing extract was evaporated under reduced pressure by using a vacuum rotary evaporator (Heidolph German) and controlled heating bath at 30 °C. The yield obtained was kept in oven at 36.5 °C for a week to remove methanol residue, then the crude extract was further purified with ethyl acetate and aqueous by using solvent partitioning techniques with modifications [18]. Two solvents (ethyl acetate and aqueous) with difference polarity were chosen to see the impact of different partitioned solvents on chemical composition and bioavailability of the *A. crispa*, since ethyl acetate is a less polar solvent and aqueous is a polar solvent. Two gram of crude hydromethanolic extract of *A. crispa* (HEAC) was soaked in 100 mL methanol, then placed in sonicator and gently mixed to dissolve the sample properly. Two hundred millilitres of sterile distilled water was added to the mixture to make a suspension. The suspension form was subjected to partition with 700 mL ethyl acetate. The two separate layers were formed and ethyl acetate and aqueous fractions were filtered and collected. The procedure was repeated twice. Then, ethyl acetate fraction was evaporated under reduced pressure by using a vacuum rotary evaporator (Heidolph German) and controlled heating bath at 30 °C. The aqueous fraction was filtered and the solution was subjected to the freeze-drying process for 4 days. The yields that obtained were stored at − 20 °C until used for analysis.

### Cell preparation and maintenance

The MCF-7, and MDA-MB-231 cancer cell lines were grown in RPMI 1640 media with L-glutamine, supplemented with 10% foetal bovine serum (FBS) (Gibco, 1% antibiotic-antimycotic (10,000 units/mL of penicillin, 10,000 μg/mL of streptomycin and 25 μg/mL amphotericin B) as a complete growth medium (CGM). All consumable materials were purchased from Gibco Thermo Fisher Scientific, USA. The cells were thawed gradually from liquid nitrogen to − 80 °C and then 36.5 °C water bath prior to culture. One millilitre of cells was transferred to 15 mL centrifuge tube and 3 mL of complete growth medium was added and centrifuged at 1200 rpm for 5 min. The supernatant was removed and pellets were re-suspended with 1 mL of complete growth medium. The 1 mL of cells suspension then was transferred to 75 cm^2^ cell culture flask. Ten mL of complete growth media was added to the flask carefully and flask was incubated at 37 °C with 5% CO_2_ in incubator. Cultured flasks were subcultured into another flask once the cells reach 80% confluency. The cells were detached with 1.5 mL of 0.25% trypsin-EDTA after removal of old media and washed with 5 mL PBS. Cells were checked microscopically daily to ensure the cells are in healthy condition.

### Microculture tetrazolium (MTT) assay

Cytotoxicity assay was prepared with seeding density of 1x10^5^cells/mL of complete growth medium in sterile 96-well flat bottom culture plates. Each well was filled with 100 μl of cells suspension (MCF 7, and MDA-MB-231). This was followed by incubation of plates at 37 °C with 5% CO_2_ overnight to allow cells attachment. Serially diluted hydromethanolic extract of *A. crispa* (HEAC), ethyl acetate extract of *A. crispa* (EAEAC), aqueous extract of *A. crispa* (AQEAC) with concentrations ranging from 15.63 μg/mL, 31.25 μg/mL, 62.5 μg/mL, 125 μg/mL, 250 μg/mL, 500 μg/mL and 1000 μg/mL, were added into the appropriate wells in four replicates for each concentration. Untreated cells (0 μg/mL) was used as control. Each concentration of treated cells, untreated cells and blank was performed in triplicate in one plate and the experiment was repeated for three times for validity. Seventy two hours treatment time was chosen which is in line with a number of previous experiment, that the treatment effect was to be done in a time dependent manner [19–21]. Therefore, following a 72-h incubation period at 37 °C with 5% CO_2_ in incubator, 20 μl of MTT solution was added to wells and incubated for an additional 4 h. Medium of each well was carefully aspirated without disturbing MTT crystal in each well. One hundred microliters of DMSO solution was added into each well to dissolve the purple formazan crystals. The optical density (OD) of formazan was proportional to the number of survival cells that metabolically active, and was read at 570 nm wavelength [22] using spectrophotometry (Infinite M200 PRO). The experiment was repeated thrice. After 4-h incubation period for formazan formation, the optical densities (OD) values, dose-response curves (percentage of cell survivability vs concentration) were generated using linear regression interpolation analysis to obtain IC_50_ (minimum concentration of hydromethanolic extract that giving 50% survival of MCF-7, and MDA-MB-231 cells). The histogram for cell survivability was constructed by using GraphPad Prism Software 5.0.

Percent of cell survivability was calculated according to the following equation:-

$$ Cell\ survivability\ \left(\%\right)=\frac{mean\  OD\  of\ treated\ cell- mean\  OD\  of\ blank}{mean\  OD\  of\ untreated\ cell- mean\  OD\  of\ blank}\times \kern0.75em 100\% $$

[23]

The cytotoxic effect against cancer cell was recorded as IC_50_ and compared with untreated cells [12, 20, 24]. The percentage of cell survivability values against concentration of respective extracts were plotted in order to determine the IC_50_ values of each extract.

### Determination of phytochemical constituents

#### Total phenolic content

Quantitative assessment of total phenolic content (TPC) was conducted according to the method described by [25, 26] by using 5 mL Folin-Ciocalteu chemical reagent diluted with 45 mL to form Folin-Ciocalteu solution. Then, 1.0 mg hydromethanolic extract of *A. crispa* (HEAC), ethyl acetate extract of *A. crispa* (EAEAC) and aquoues extract of *A. crispa* (AQEAC) were dissolved in 99.9% assay percent range of methanol, purchased from Fisher Scientific, USA. Each 100 μL of extract solution, control solution (methanol) and standard (gallic acid) were mixed with 400 μL of 7.5% sodium bicarbonate (NAHCO_3_) and 500 μL Folic-Ciocalteu solutions. The mixture was incubated for 2 h in the dark room at 40 °C. All the tested samples (200 μL) were pipetted into 96 well ELISA plate readers. Optical density (OD) was determined at 760 nm. Gallic acid calibration curve was generated from the gallic acid – Folin reaction ranging from 100 μg/mL, 50 μg/mL, 25 μg/mL, 12.5 μg/mL, 6.25 μg/mL, and 3.13 μg/mL. The results were expressed as milligram (mg) gallic acid per one gram extract (mg GAE/g extract).

#### Total flavonoid content

Quantitative assessment of total flavonoid content (TFC) was conducted in line with the method described by [25, 26]. Basically, 150 μL from (1 mg/mL methanol) of HEAC, EAEAC and AQEAC was mixed with 150 μL 10% aluminium chloride (AlCl_3_) solution and incubated for 10 min at dark room. Meanwhile, 150 μL methanol was used as negative control and 150 μL of rutin (100 μg/mL, 50 μg/mL, 25 μg/mL, 12.5 μg/mL, 6.25 μg/mL, 3.13 μg/mL) were used as standard. Both are mixed with 150 μL of 10% AlCl_3_ and incubated similar to the extracts. Three hundred microliter of each extract, negative control and standard mixtures were pipetted out into 96 well plates and analyzed under spectrophotometer at 435 nm. Rutin calibration curve was generated from the optical density of rutin – AlCl_3_ reaction ranging from 100 μg/mL, 50 μg/mL, 25 μg/mL, 12.5 μg/mL, 6.25 μg/mL, and 3.13 μg/mL. The results were expressed as milligram (mg) rutin per one gram extract (mg RE/g extract).

#### Antioxidant assays

##### DPPH free radicals scavenging assay

Antioxidant test by using DPPH (1,1-diphenyl-1-picrylhydrazyl) compound was conducted according to the method described by [27]. Four point two milligram of DPPH compound was diluted with 50 mL of methanol to make a DPPH solution then, the chemical was incubated for 2 h. One milligram of trolox was dissolved into one milliliter of methanol as a stock solution. Trolox (6-hydroxyl-2,5,7,8-tetramethyichroman-2-carboxylic acid, 97% purity) was used as reference (standard). Then, one hundred microliter of trolox solution was serially diluted into final concentrations which were 50 μg/mL, 25 μg/mL, 12.5 μg/mL, 6.25 μg/mL, and 3.13 μg/mL. Methanol served as negative control while distilled water is the blank. Each of the negative control and standard were mixed with 585 μL DPPH solution in 2 mL vial and wrapped with aluminum foil. The tested samples were incubated in the dark room for one hour.

The tested samples were analyzed simultaneously by using spectrophotometer at 515 nm wavelength and performed triplicate (*n* = 3) for effectual reproducibility. Effective concentration of trolox that providing 50% antioxidant capacity (EC_50_) was calculated according to the formula stated below to obtained trolox calibration curve:

$$ \mathrm{Scavenging}\ \mathrm{activities}\ \left(\%\right):\left({\mathrm{A}}_{\mathrm{c}}-{\mathrm{A}}_{\mathrm{s}}\right)/\left({\mathrm{A}}_{\mathrm{c}}\right)\times 100\% $$

where:

A_c_: Absorbance of control.

A_s_: Absorbance of sample.

The antioxidant capacity of HEAC, EAEAC, and AQEAC against the DPPH solution then was estimated through Trolox calibration curve. The results were expressed as milligram Trolox equivalent antioxidant capacity (TEAC) per one gram of tested extract.

##### ABTS free radical scavenging assay

Antioxidant test using ABTS (2,2′-azino-bis(3-ethylbenzothiazoline-6-sulphonic acid) compound was conducted according to the method described by [27]. ABTS stock solution was prepared by mixing 2.45 mM potassium persulphate (K_2_S_2_O_8_) solution with 38.4 mg/mL ABTS in a volumetric flask and incubated for 16 h in dark room. The mixture then was continually diluted with double distilled water and monitored spectrophotometrically at 735 nm until the absorbance value was at 0.7.

Trolox (1mg/mL) was prepared as a stock solution. Then, one hundred microliter of Trolox solution was serially diluted into final concentrations which were 100 μg/mL, 50 μg/mL, 25 μg/mL, 12.5 μg/mL, 6.25 μg/mL, and 3.13 μg/mL. While trolox solution acted as a reference for antioxidant capacity, methanol served as a negative control with distilled water being the blank. Each of negative control and standard were mixed with 2.85 mL ABTS solution in 15 mL centrifuge tube and wrapped with aluminum foil to prevent light penetration. The mixtures were incubated in the dark room for one hour and organized into 96 well plates. Each concentration was performed in triplicate (*n* = 3) for effectual reproducibility. The plate was analyzed by using spectrophotometer at 734 nm wavelength. In order to obtain trolox calibration curve, effective concentration of trolox that providing 50% antioxidant capacity (EC_50_) was calculated according to the formula stated below:

$$ \mathrm{Scavenging}\ \mathrm{activities}\ \left(\%\right):\left[\left({\mathrm{A}}_{\mathrm{c}}-{\mathrm{A}}_{\mathrm{s}}\right)/\left({\mathrm{A}}_{\mathrm{c}}\right)\right]\times 100\% $$

where:

A_c_: Absorbance of control.

A_s_: Absorbance of sample.

The antioxidant capacity of HEAC, EAEAC, and AQEAC against ABTS free radical solution then, was estimated through Trolox calibration curve. The results were expressed as milligram trolox equivalent antioxidant capacity (TEAC) per one gram of tested extract.

### Statistical analysis

All the percentages of cell survivability were expressed as mean (*n* = 3) per plate ± SD (Standard deviation) and differences among treated and untreated cells were analysed using one way ANOVA followed Dunnett’s multiple comparison test. The test was considered statistically significant when *P* < 0.05 as compared to untreated cell (control) and GraphPad Prism Software 5.0 was used to analyse all statistical tests.

Data were expressed as mean of three times experiment (n = 3) ± SD (standard deviation) and the difference of TPC and TFC among sample extracts were determined using one way analysis of variance (ANOVA) followed by Tukey’s multiple comparison test. The statistical analysis test was performed using GraphPad Prism Software 5.0. The sample was differred significantly when (*P* < 0.05).

## Results

### Effect of HEAC, EAEAC, and AQEAC on MCF-7 and MDA-MB-231 breast cancer

**Fig. 2:**
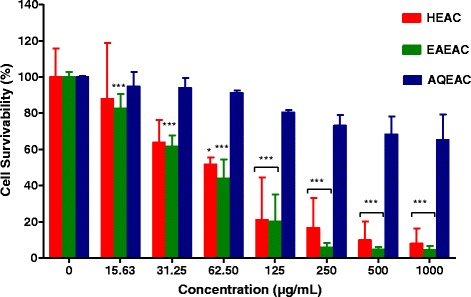
Cell survivability (%) of MCF-7 breast cancer cell line following treatment with HEAC, EAEAC and AQEAC extracts for 72 h. All values are expressed as mean (*n* = 3) ± SD for triplicate. The comparison between treated cells and untreated cells was evaluated using one way ANOVA followed by Dunnett’s multiple comparison test. * *P* < 0.05, and *** *P* < 0.001 denote significant difference as compared to untreated cell (control)

**Fig. 3:**
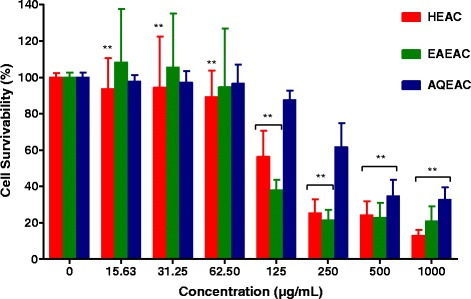
Cell survivability (%) of MDA-MB-231 breast cancer cell line following treatment with HEAC, EAEAC, AQEAC extracts for 72 h. All values are expressed as mean (n = 3) ± SD for triplicate. The comparison between treated cells and untreated cells was evaluated using one way ANOVA followed by Dunnett’s multiple comparison test. **P < 0.01, denote significant difference as compared to untreated cell (control)

Figures 2 and 3 show the cell survivability (%) of MCF-7 and MDA-MB-231 breast cancer cell line following treatment with HEAC, EAEAC and AQEAC extracts for 72 h, respectively. The IC50 values of HEAC, EAEAC and AQEAC were shown in Table 1. In general, AQEAC showed a poor cytotoxic effect on breast cancer cell lines with IC50 values more than 300 μg/mL on both breast cancer cell lines (Table 1).

**Table 1:** IC50 values of HEAC, EAEAC and AQEAC in breast cancer cell lines

Cancer cell lines	IC_50_ values of extracts/drug (μg/mL)		
	HEAC	EAEAC	AQEAC
MCF-7	57.35 ± 19.33	54.98 ± 14.10	> 1000
MDA-MB-231	139.57 ± 31.64	108.13 ± 11.23	347.44 ± 98.78

### Quantitative determination of total phenolic contents (TPC)

**Fig. 4:**
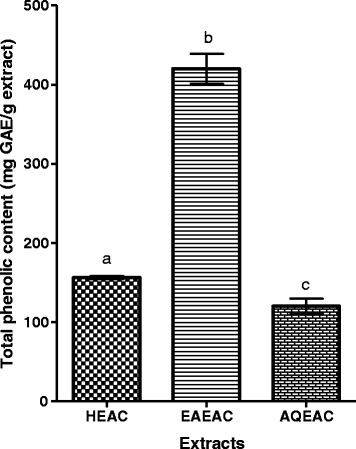
Total phenolic content of the HEAC is 156.30 ± 2.00, EAEAC is 419.92 ± 18.76 and AQEAC is 120.32 ± 9.54 mg GAE/g extract, respectively. Data for total phenolic content are expressed in means (three replicates) ± SD. Means with different letter within the same column differ significantly (*P* < 0.05) according to one way ANOVA following Tukey’s multiple comparison test

The results of total phenolic content from HEAC, EAEAC and AQEAC were shown in Fig. 4 ranging from 120.32 to 419.92 mg GAE/g extract. Overall, *A. crispa* contain very high amount of phenolic compound. Based on standard a TPC value higher than 10 mg GAE/g extract was considered high [28]. However, amongst EAEAC extract has the highest amount of total phenolic content followed with HEAC and AQEAC (Fig. 4). The TPC values was obtained from gallic acid calibration curve (y = 0.002× – 0.0004; R^2^ = 0.999).

### Quantitative determination of total flavonoid contents (TFC)

**Fig. 5:**
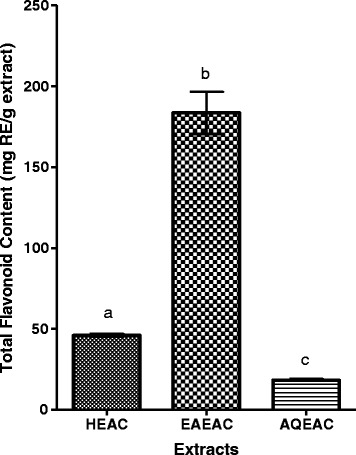
Total flavonoid content of the HEAC is 45.93 ± 0.94, EAEAC is 183.62 ± 13.10 and AQEAC is 18.32 ± 0.94 mg RE/g extract, respectively. Data for total flavonoid content are expressed in means (three replicates) ± SD. Means with different letter within the same column differ significantly (*P* < 0.05) according to one way ANOVA following Tukey’s multiple comparison test

The flavonoid content of HEAC, EAEAC and AQEAC were expressed as rutin equivalents (RE). From the results shown in Fig. 5, *A. crispa* contain very high amount of flavonoid in EAEAC and followed by HEAC and AQEAC. TFC was considered high when its amount more 10 mg RE/g extract. The TFC values were obtained from rutin calibration curve (y = 0.0117× + 0.0177; R^2^ = 0.9989).

### Antioxidant capacities

Antioxidant capacities of hydromethanolic extract of *A. crispa* and its partitions, namely EAEAC and AQEAC were assessed by 2 types of antioxidant tests, including DPPH and ABTS assays. The total content of phenolic and it derivatives, flavonoid from all HEAC, EAEAC and AQEAC were also been evaluated to link the relationship.

The DPPH and ABTS radicals scavenging activities were estimated through Trolox calibration curve (y = 1.0429×-0.2747; R^2^ = 0.9999) and (y = 0.5167×-0.3429; R^2^ = 0.9996), respectively. The tested extracts were performed in triplicate (*n* = 3) and was expressed as milligram (mg) Trolox equivalent antioxidant capacity (TEAC) per 1 g (g) of tested extract (Table 2 and Table 3). Overall, antioxidant capacity of *A. crispa* was considered high. HEAC, EAEAC and AQEAC showed good scavenging activities, able to scavenge at least 50% of free radicals solution below than 10 mg/mL of extract concentration.

**Table 2:** DPPH scavenging assay of *A. crispa* leaves from different solvent extracts

Extract	Antioxidant capacity (mg TEAC/g extract)
HEAC	175.64 ± 2.89
EAEAC	416.28 ± 2.46
AQEAC	89.24 ± 1.67

The tested extract were performed triplicate (n = 3) and was expressed as milligram (mg) Trolox equivalent antioxidant capacity (TEAC) per 1 g (g) of tested extract

**Table 3:** ABTS scavenging assay of *A.crispa* leaves from different solvent extracts

Extract	Antioxidant capacity (mg TEAC/g extract)
HEAC	294.62 ± 10.14
EAEAC	1228.68 ± 65.19
AQEAC	186.02 ± 3.83

The tested extract were performed triplicate (n = 3) and was expressed as milligram (mg) Trolox equivalent antioxidant capacity (TEAC) per 1 g (g) of tested extract

## Discussion

The cytotoxic effect of HEAC and its partitions were assessed based on the minimum concentration of extract that giving at least 50% of the cancer cell survivability (IC_50_). The four categories of extracts which are; very active (IC_50_ ≤ 20 μg/mL), moderately active (IC_50_ > 20–100 μg/mL), weakly active (IC_50_ > 100–1000 μg/mL) and inactive (IC_50_ > 1000 μg/mL), [23, 29]. For pure compound or drug, IC_50_ value less than 4 μg/mL is considered potent [30, 31].

Cytotoxic analysis revealed that, HEAC and EAEAC possessed moderate cytotoxic effect against MCF-7 with IC_50_ 57.35 ± 19.33 μg/mL and 54.98 ± 14.10 μg/mL, respectively. HEAC and EAEAC showed weak cytotoxic effect (IC_50_ > 100–1000 μg/mL) on MDA-MB-231. For AQEAC, the IC_50_ value against MCF-7 was more than 1000 μg/mL and MDA-MB-231 was 347.44 ± 98.78 μg/mL indicating that AQEAC has poor cytotoxic effect against breast cancer. In this study, it showed the response of breast cancer cell lines toward *A. crispa* extract and its partitions was variable. It is in agreement with previous studies that the response towards each breast cancer was difference depending on the classification and degree of malignancy of cancer cells [32]. Moreover, differences in cell line, plant extract, solvent used, and plant source also contribute to the difference cytotoxic effect possessed by the plant [33]. However, from the results, it might suggest that oestrogen receptor (ER) positive breast cancer susceptible to hydromethanolic and ethyl acetate extracts of *A. crispa*.

Overall, *A. crispa* plant had high TP, TFC values and antioxidant capacity. However, amongst extracts, EAEAC had the highest level of TPC, TFC and antioxidative activities followed by HEAC and AQEAC. HEAC and EAEAC revealed more total phenolic and flavonoid contents as compared to aqueous extract (AQEAC). This result explained the weak cytotoxicity effect exhibited by AQEAC as the phytochemical compounds contribute significantly in the ethnopharmacological medicinal values of the plants.

It is also revealed from the analysis conducted that EAEAC possessed the highest scavenging activities as high antioxidative activities were contributed by the highest level of TPC and TFC values of the extarct. Ethyl acetate might be the good solvent for extracting phytochemical compounds with antioxidant properties. This is similar to previous studies conducted by [34], that TPC of ethyl acetate (EA) extract of *Alpinia mutica* (1.55 ± 0.16 mg GAE/g extract) was the highest when compared to its crude hydromethanolic, hexane and aqueous extract. The results from the same study also revealed that EA of *Alpinia mutica* possessed the highest antioxidant capacity (EC_50_: 0.125 ± 0.04 μg/mL) which correlates with its TPC finding. TPC was considered high when TPC level higher than 10 mg GAE/g extract [28]. The level of TFC and TPC are the same in descending order; EAEAC > HEAC > AQEAC because flavonoid is a subgroup of phenolics compound.

For antioxidant capacity determination, *A. crispa* possess high antioxidant capacity which is good to scavenge free radical in the body. The benchmark for the plant was considered high TPC and TFC values 10 mg GAE/g extract and 10 mg RE/ g extract, respectively, while for DPPH and ABTS scavenging assays were EC_50_ < 10 mg/mL [26, 35]. EAEAC contains highest TPC and TFC followed with HEAC and AQEAC. The findings of DPPH and ABTS scavenging assays also showed that *Ardisia crispa* leaves extract possess high antioxidant capacity and the results were in agreement with TPC and TFC findings. EAEAC has the highest scavenging activity followed by HEAC and AQEAC. These results in agreement with many previous studies who found that the level of TPC and TFC in extract play a major role for antioxidant capacity [36–39]. The difference of antioxidant capacities among HEAC, EAEAC and AQEAC was due to the solvent used during partitioning. The change in location of hydroxyl group attached to aromatic ring in phenolics compound and benzene ring of flavonoids specifically affect the antioxidant properties [40, 41].

Notwithstanding that the EAEAC exhibit the highest antioxidative activity, it is not a potent cytotoxic plant. This is because, not necessarily phenolic and flavonoid compounds are responsible for anticancer properties. The phytochemical compound of plants are of multicomponent mixture. Other phytochemical constituents such as saponins also exhibit anticancer properties [42, 43]. Besides, phenolic has broad secondary plant metabolites and many subgroup/class. Moreover, it can presence with combination of other compounds such as terpenoids, saponins, glycosides, chlorophyl, lipid, protein, polysaccharides and cyanides [44]. For example, terpenoids and saponins are phytochemical compounds that have anticancer effect [45–47]. Hence, it is plausible that during partitioning of HEAC with ethyl acetate and aqueous solvent, the chemical characteristic or solubility of some phytochemical composition was altered, thus making it toxic or less potent. Since, phytochemical compound is a complex multicomponent form, separation of the compounds possibly alter the action of mixture compound, resulting in the loss of synergestic effect of the extract. Higher phenolic and flavonoid compounds in EAEAC as compared to HEAC and AQEAC, could be due to some phenolic subgroup/subclass reacts strongly with the Folin–Ciocalteu reagent [48, 49]. Therefore, the use of solvent partitioning method might contribute to structural alteration effect of the phytochemical compounds and affect the therapeutic value. Based on reference value and other studies, HEAC is considered among extracts that have good scavenging activitiy. Futhermore, HEAC is in the same category of cytotoxic effect as EAEAC against MCF-7 and MDA-MB-231. The only criteria that need to be considered to choose among HEAC and EAEAC is the safetly level. Extracts with good scavenging activities might be beneficial in complimentary with chemotherapeutic drugs in order to minimizing the side effects from the chemotherapeutic agent. In addition, it can perhaps prevent the carcinogenesis pathway.

## Conclusion

Based on results, hydromethanolic and ethyl acetate extracts of *Ardisia crispa* can be a potential candidate for oestrogen receptor (ER) positive breast cancer agent because it is more cytotoxic to MCF-7 cancer cell lines as compared to MDA-MB-231. Thus, the extracts are believed to exert good scavenging activities via phenolic and flavonoid compounds. All these findings indicate the need for new investigations with blocker of oestrogen receptor (ER) with the aim to strictly define the anti-oestrogenic effect of *Ardisia crispa* extracts on ER positive cancer cells. The successful results of this, an additional set of investigations, after in vivo studies, could help to understand is it possible to combine the most powerful extracts with the available drug and to encounter drug resistance and adverse effects.

## Abbreviations

ABTS: 2, 2′-azinobis (3-ethylbenzothiazoline-6-sulphonic acid)
AQEAC: Aqueous extract of *Ardisia crispa*
DMSO: Dimethyl Sulphoxide
DPPH: (1,1-diphenyl-2-picrylhydrazyl) radical scavenging assay
EAEAC: Ethyl acetate extract of *Ardisia crispa*
FBS: Foetal bovine serum
HEAC: Hydromethanolic extract of *Ardisia crispa*
MTT: Microculture tetrazolium (3-(4,5-dimethylthiazol-2-yl)-2,5-diphenyltetrazolium bromide) assay
TFC: Total Flavonoid Content
TPC: Total Phenolic Content
UPM: Universiti Putra Malaysia
WHO: World Health Organization
